# Odours of *Plasmodium falciparum*-infected participants influence mosquito-host interactions

**DOI:** 10.1038/s41598-017-08978-9

**Published:** 2017-08-24

**Authors:** Jetske G. de Boer, Ailie Robinson, Stephen J. Powers, Saskia L. G. E. Burgers, John C. Caulfield, Michael A. Birkett, Renate C. Smallegange, Perry J. J. van Genderen, Teun Bousema, Robert W. Sauerwein, John A. Pickett, Willem Takken, James G. Logan

**Affiliations:** 1 Laboratory of Entomology, Wageningen University, Droevendaalsesteeg 1, 6708 PB Wageningen, The Netherlands; 2 London School of Hygiene and Tropical Medicine, Keppel Street, London, WC1E 7HT United Kingdom; 3 Computational and Systems Biology Department, Rothamsted Research, Harpenden, Hertfordshire AL5 2JQ United Kingdom; 4 Biometris, Wageningen University, Droevendaalsesteeg 1, 6708 PB Wageningen, The Netherlands; 5 Biological Chemistry and Crop Protection Department, Rothamsted Research, Harpenden, Hertfordshire AL5 2JQ United Kingdom; 6 Institute for Tropical Diseases, Harbour Hospital Rotterdam, Haringvliet 72, 3011 TG Rotterdam, The Netherlands; 7 Medical Microbiology, Radboud University Medical Centre, Geert Grooteplein 26-28, 6525 GA Nijmegen, The Netherlands; 8 Present Address: Netherlands Institute of Ecology, Droevendaalsesteeg 10, 6708 PB Wageningen, The Netherlands

## Abstract

Malaria parasites are thought to influence mosquito attraction to human hosts, a phenomenon that may enhance parasite transmission. This is likely mediated by alterations in host odour because of its importance in mosquito host-searching behaviour. Here, we report that the human skin odour profile is affected by malaria infection. We compared the chemical composition and attractiveness to *Anopheles coluzzii* mosquitoes of skin odours from participants that were infected by Controlled Human Malaria Infection with *Plasmodium falciparum*. Skin odour composition differed between parasitologically negative and positive samples, with positive samples collected on average two days after parasites emerged from the liver into the blood, being associated with low densities of asexual parasites and the absence of gametocytes. We found a significant reduction in mosquito attraction to skin odour during infection for one experiment, but not in a second experiment, possibly due to differences in parasite strain. However, it does raise the possibility that infection can affect mosquito behaviour. Indeed, several volatile compounds were identified that can influence mosquito behaviour, including 2- and 3-methylbutanal, 3-hydroxy-2-butanone, and 6-methyl-5-hepten-2-one. To better understand the impact of our findings on *Plasmodium* transmission, controlled studies are needed in participants with gametocytes and higher parasite densities.

## Introduction

Parasitic manipulation of hosts is found commonly, and is likely to have far-reaching consequences for disease ecology and epidemiology e.g.^1^. For vector-transmitted parasites of vertebrates, transmission requires contact between the vector and the host, usually by blood feeding. Natural selection will favour parasite genotypes that influence the phenotype of their host in such a way that this contact rate is enhanced and transmission increased^2, 3^. Parasite-induced effects on feeding and host-seeking behaviour of hematophagous vectors are known from various systems, including *Plasmodium*-infected mosquitoes e.g.^4–6^. Parasites also influence vertebrate host phenotypes, for example, *Trypanosoma* parasites influence the uptake of blood from cows by tsetse flies^7–9^ and it has been suggested that *Leishmania* parasites increase attractiveness of hamsters to sandfly vectors^10^.

It is thought that malaria parasites may influence attractiveness of the vertebrate host, including humans, to *Anopheles* mosquitoes. Kenyan children infected with gametocytes (transmissible stages) of *P. falciparum* attracted twice as many mosquitoes than children with asexual parasites or children without parasites^11^. More recently, adult humans carrying *P. vivax* gametocytes were suggested to attract higher numbers of *Anopheles darlingi* mosquitoes than infected persons without gametocytes^12^. Host odour is known to be particularly important for host-seeking mosquitoes e.g.^13–15^, and has been suggested to be a likely target of manipulation by malaria parasites^16, 17^. For example, in the *P. chabaudii* model system, mosquito attraction was enhanced during the chronic infection stage when mice were highly infectious but acute symptoms had subsided^16^. Overall, volatile emission was increased during infection and individual compounds associated with infection were identified. Hexanoic acid, 2- and 3-methylbutanoic acid, and tridecane each individually increased attraction of *An. stephensi* when added to the odour of healthy mice^16^. Conversely, benzothiazole, which was suppressed during infection, reduced attraction of mosquitoes when added to the odour of infected mice.

In humans, *P. falciparum* infection was recently shown to affect concentrations of several chemicals in breath^18^. Levels of thioethers were correlated with parasitaemia and are promising biomarkers of infection. However, breath is thought to play a small role in mosquito attraction compared to skin odour^19^. In the present study, we therefore investigated whether *P. falciparum* infection affected skin odour profile of human participants in Controlled Human Malaria Infections (CHMI) and host-seeking behaviour of *An. coluzzii* mosquitoes.

## Results and Discussion

### Sample collection and *P. falciparum* infection status

Participants were recruited in two clinical CHMI studies where *P. falciparum* was delivered through bites of infectious *An. stephensi* mosquitoes^20^ (further referred to as CHMI1 and CHMI2). Skin odour samples were collected two days before challenge with *P. falciparum* (referred to as Before), during infection (6 and/or 8 days post challenge, During), and post-treatment (34 days post challenge, After). Of the 17 participants, 12 became qPCR-positive for *P. falciparum* during sample collection [During (+)]. During (−) individuals were not parasitologically positive at any sampling timepoint (Supplementary Fig. S1). Mature gametocytes were not detected in any of the participants on the day of antimalarial treatment, and the following days.

### Human odour profiles

**Figure 1:**
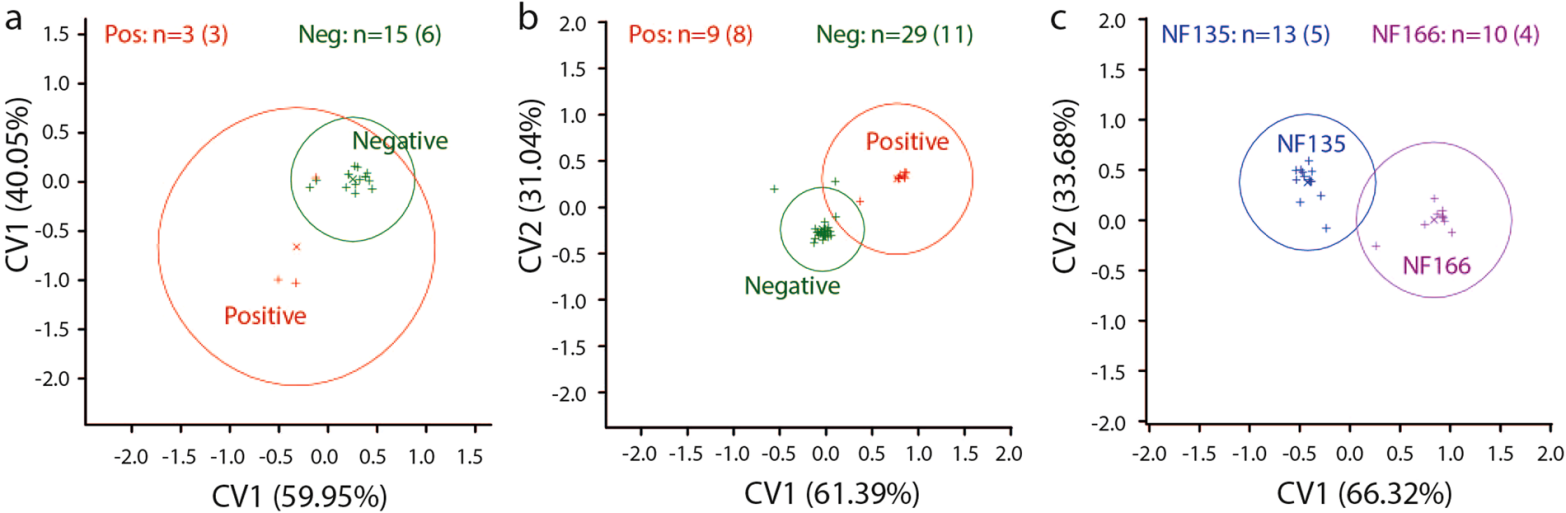
Effect of *Plasmodium falciparum* infection on the composition of human skin odour. Canonical variates plots of (**a**) CHMI1 and (**b**) CHMI2 chemistry data (Porapak samples) are grouped by parasitological status (irrespective of sampling timepoint), and (**c**) grouped by *P. falciparum* strain within samples of CHMI2 (numbers of samples per group with number of individuals in brackets). CHMI1 and CHMI2 refer to two controlled human malaria infection studies in which our samples were collected. Participants were infected with *P. falciparum* strain NF54 in CHMI1, and with strains NF135.C8 or NF166.C10 in CHMI2. Samples are positioned by scores (indicated by + symbols) on canonical variate axes relative to the presence and quantity of compounds contributing to these axes. The percentage of possible discrimination accounted for is included in the axes labels. Circles represent 95% confidence intervals around means (x symbols) of scores. Controls of empty bag sampling and the diethyl ether used during sample elution were analysed and included as control groups but excluded from figures to maintain clarity. Parasitological status was defined by qPCR-testing on the same day. In panel C, samples collected during and after infection are included from participants that became parasitologically positive at least once during infection.

Both total volume and the constituent compounds (odour profile) of skin volatiles from CHMI participants were affected as early as 6–8 days after *P. falciparum* challenge, corresponding to on average 2 days after the parasites move from the liver to the blood. There was a trend for greater total amount of all compounds in parasitologically positive compared to negative samples in both series (Supplementary Fig. S2). Compound presence/absence and quantity were compared using canonical variate analysis (CVA) to identify compounds that contributed to the separation of groups. For both CHMI studies, good separation was achieved using parasitological status as the grouping factor, despite the small sample size which led to overlapping 95% confidence intervals (Pos vs. Neg, Fig. 1a and b). CVA using temporal *P. falciparum* infection status in the grouping of samples [Before, During (−), During (+), After], indicated almost the same set of compounds, with some additional peaks that mostly contributed to separation of post-infection status in CHMI2 (After, Supplementary Fig. S3).

**Figure 2:**
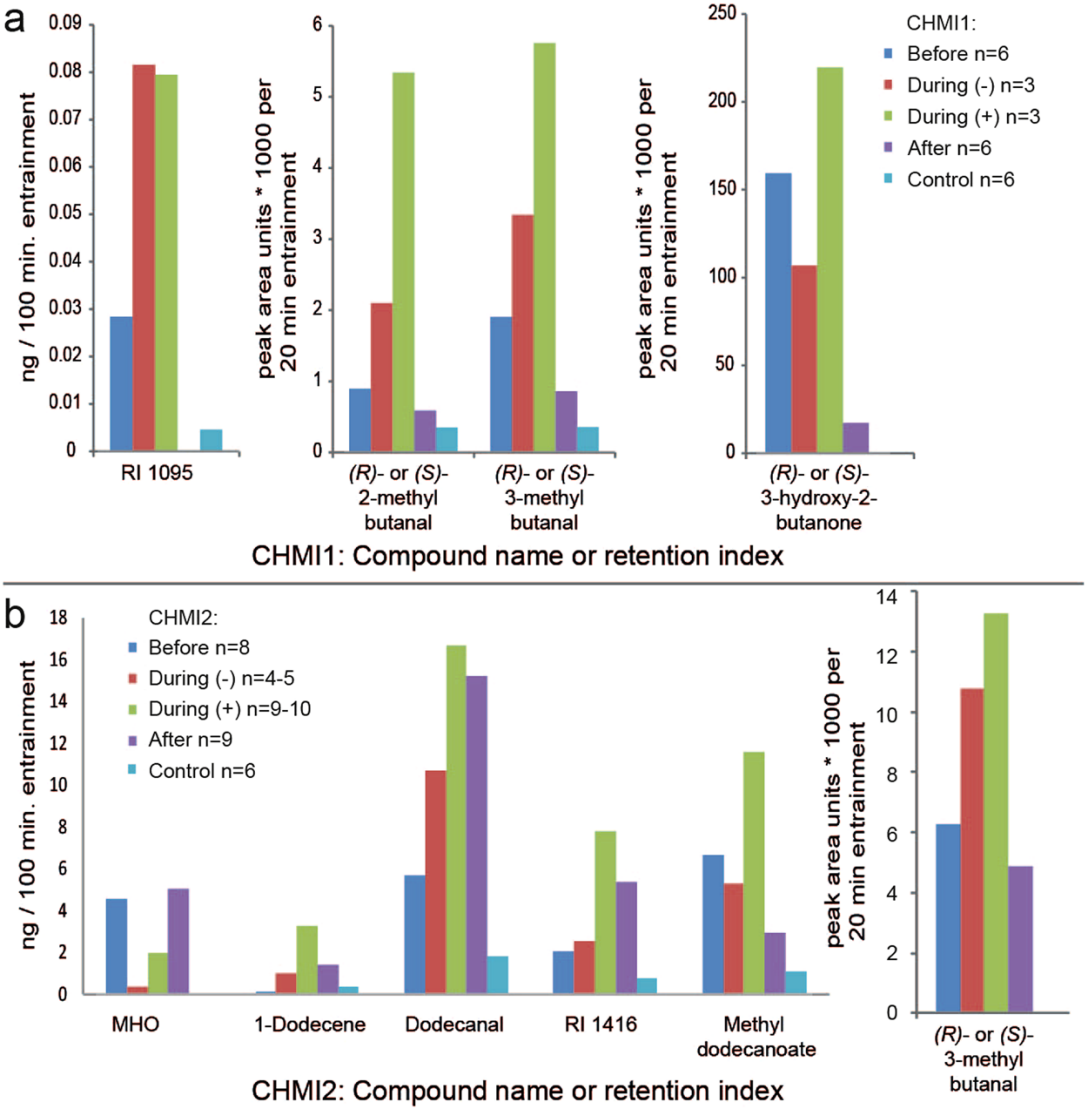
Effect of *Plasmodium falciparum* infection on levels of specific human volatile compounds. Compounds of interest identified in samples of (**a**) CHMI1 and (**b**) CHMI2 were analysed by temporal parasitological status [Before: sampling timepoint before malaria challenge; During: 6–8 days post malaria challenge, with (−) and (+) referring to parasitological status, measured by qPCR; After: after antimalarial treatment; Control: headspace collected from empty control bags]. Back-transformed predicted means (REML) are shown (amounts in ng collected over 100 min. for Porapak – left two panels, amounts in peak area units*1000 collected over 20 min for Tenax – right three panels). For predicted means (log transformation) with standard errors, see Supplementary Fig. S4 and Tables S1 and S2 for pairwise SEDs. In CHMI2, the number of Porapak and Tenax samples varied as indicated in the figure legend; exact n given in Table 2, and Fig. S4. RI 1095 (CHMI1) and 1-Dodecene, dodecanal, methyl dodecanoate and RI 1416 (CHMI2) each co-eluted with another unidentified compound and hence their quantities are difficult to obtain accurately. Differences in amounts observed in temporal *P. falciparum* infection categories can therefore not be attributed with absolute certainty to these compounds.

Several volatile compounds [tentatively identified by coupled gas chromatography-mass spectrometry (GC-MS)] appeared to characterize *P. falciparum* challenged individuals or a more general ‘infection’ profile, mostly by increased production in parasitologically positive samples. Compounds of likely biological origin were regarded as compounds of interest. Over the two CHMI studies, nine compounds were found to vary substantially between certain groups (Fig. 2 and Tables 1 and 2; see also Supplementary Fig. S4 and Tables S1 and S2), including *(R)-* or *(S)-*2-methylbutanal, *(R)-* or *(S)-*3-methylbutanal, *(R)-* or *(S)-*3-hydroxy-2-butanone, 6-methyl-5-hepten-2-one, a dodecene (most likely 1-dodecene, based on retention time), dodecanal and methyl dodecanoate Inspection of GC-MS data of retention index (RI) 1095 showed two compounds of interest. These were *(R)*- or *(S)*-2-ethylhexanoic acid, which are likely human volatile products, and another unidentified compound. The peak at RI 1416 included a compound that was tentatively identified as a sesquiterpene, and a phthalate, which is considered to be of exogenous origin (Table 2). None of the *P. falciparum* infection-associated compounds identified on the breath by Berna *et al*. were found in this study^18^, suggesting that early stages and low densities of *P. falciparum* infection can induce a range of physiological changes in the human body that affect breath and body odour differentially.

**Table 1:** Compounds of interest (COI) derived from CHMI1, with significant pairwise differences between temporal parasitological groups (LSD, P < 0.05).

COI (RI^a^):	(Tentative) identification	CAS-number	Av. SED^b^	P, DF^b^	Significant pairwise differences^c,d^
1095	2-ethyl hexanoic acid^e^	149-57-5	0.734	*P* = *0.028*, 13	After vs Before, During (−) and During (+)
					Control vs. During (−) and During (+)
Tenax	2-methylbutanal	96-17-3	0.672	*P* = *0.009*, 11	During (+) vs. Before, After and Control
					During (−) vs After
Tenax	3-methylbutanal	590-86-3	0.728	*P* = *0.031*, 11	During (+) vs After and Control
					During (−) vs After and Control
Tenax	3-hydroxy-2-butanone	513-86-0	1.071	*P* < *0.001*, 12	Control vs. all other groups
					After vs. Before

^a^Retention indices (RI) are given for compounds detected on Porapak samples.

^b^Average standard error of the difference, P-value and degrees of freedom (DF) derived from linear mixed models, which were fitted using the method of residual maximum likelihood.

^c^Number of samples in each group (number of individuals in brackets): Before n = 6 (6), During (−) n = 3 (3), During (+) n = 3 (3), After n = 6 (6), (Empty bag) Control n = 6.

^d^Full matrices of SEDs including results of pairwise comparisons made per compound are provided in Supplementary Table S1.

^e^Another unidentified compound co-eluted at the same retention time.

**Table 2:** Compounds of interest derived from CHMI2, with significant pairwise differences between temporal parasitological groups (LSD, P < 0.05).

COI (RI^a^)	(Tentative) identification	CAS number	Av SED^b^	P, DF^b^	Significant pairwise differences^c,d^
972	6-methyl-5-hepten-2-one	110-93-0	0.452	*P* = *0.002*, 24	During (−) vs. Before and After
					Control vs. Before, During (+) and After
1193	1-Dodecene^e^	112-41-4	0.512	*P* = *0.050*, 24	Before vs. During (+)
1392	Dodecanal^e^	112-54-9	0.404	*P* < *0.001*, 32	Before vs. During (+) and After
					Control vs. all groups
1416	Sesquiterpene^e^	n/a	0.532	*P* = *0.031*, 32	During (+) vs. Before
					Control vs. During (+) and After
1509	Methyl dodecanoate^e^	111-82-0	0.621	*P* = *0.076*, 24	During (+) vs. After
					Control vs. During (+)
Tenax	3-methylbutanal	590-86-3	0.847	*P* < *0.001*, 25	During (+) vs. After

^a^Retention indices (RI) are given for compounds detected on Porapak samples.

^b^Average standard error of the difference, P-value and degrees of freedom (DF) derived from linear mixed models, which were fitted using the method of residual maximum likelihood.

^c^Number of Porapak samples in each group (number of individuals in brackets): Before n = 8 (8), During (−) n = 5 (5), During (+) n = 9 (8), After n = 9 (9), (Empty bag) Control n = 6. Number of Tenax samples in each group: Before n = 8 (8), During (−) n = 4 (4), During (+) n = 10 (9), After n = 9 (9), (Empty bag) Control n = 6.

^d^Full matrices of SEDs including results of pairwise comparisons made per compound are provided in supplementary Table S2.

^e^A co-eluting compound was detected at the same retention time.

The ketone 6-methyl-5-hepten-2-one (MHO) was present in lower amounts in During (−) samples in CHMI2 compared to Before or After samples (least significant difference, LSD, P < 0.05; Fig. 2b), while levels in During (+) samples were statistically similar to those in Before and After samples. The level of MHO was unaffected in two participants that remained parasitologically negative throughout the study and considered to be non-immune challenged, suggesting that the observed down-regulation of MHO emission in During (−) samples is associated with infection. This is interesting because MHO is known to affect mosquito behaviour^21^. Although reported as a weak attractant in one study^22^, MHO can inhibit flight responses of *Aedes aegypti* mosquitoes^23^, and high concentrations are repellent against *Ae. aegypti*, *An. gambiae s.s*. and *Culex quinquefasciatus* ^24, 25^. It will be important to further establish the role of MHO as part of the skin volatile profile of healthy and *P. falciparum* infected humans.

We found an increase in the amount of an unidentified sesquiterpene (RI 1416) in *P. falciparum* challenged individuals, although a phthalate co-eluted at the same retention time (LSD, P < 0.05, Fig. 2b). Terpenes are often considered to be of exogenous origin when found in human odour^26^, but were recently reported in the headspace of cultured malaria parasites in two studies^27, 28^. It was suggested that these compounds may be emitted directly from the parasites themselves, via exhaled breath and subsequently influence mosquito behaviour, and it is possible that these compounds are also released through the skin, although they were not found in association with *P. chabaudii*-infected mice^16^.

Several compounds that are known to be produced by bacteria on human skin^29^ were associated with *P. falciparum* infection in our study (REML, P < 0.031, Fig. 2, Tables 1 and 2; and see Supplementary Fig. S4 and Tables S1 and S2). In CHMI1, both 2-methylbutanal and 3-methylbutanal were found in significantly greater amounts in During (+) and During (−) samples than in After samples. In CHMI2, this effect was most pronounced for 3-methylbutanal, which was also present in significantly greater amounts in During (+) than in After samples (LSD, P < 0.05). The ratio of isomers [*(R)-* or *(S)*] of these compounds was not determined. These compounds are important candidates in mediating differential attraction to *P. falciparum* infected individuals, as they are known to be attractive to *An. coluzzii* ^30^, and skin bacteria play an important role in differential attractiveness between healthy human individuals^15^. Our findings therefore suggest that *P. falciparum* may change the human odour profile by influencing skin microbial communities, perhaps through modulation of the immune system, and we are currently investigating this.

1-Dodecene and dodecanal were present in significantly greater amounts in During (+) samples than in samples collected before *P. falciparum* challenge (LSD, P < 0.05; Table 2, Fig. 2b) but this effect was not seen in During (−) samples, suggesting an association with the presence of parasites. In After samples, the amount of 1-dodecene returned to statistically similar levels to Before samples, while amounts of dodecanal continued to be greater in After than in Before samples.

The effect of temporal parasitological status on emission levels of methyl dodecanoate was marginally insignificant (P = 0.076, Table 2), but pairwise comparisons showed that it was present in significantly greater amounts in During (+) samples than in After samples (LSD, P < 0.05; Fig. 2b). Additionally, amounts of methyl dodecanoate in During (+) samples were significantly greater than in control samples without human skin entrainment, while Before, During (−) and After samples did not differ from controls, suggesting that these changes may have been induced by the presence of *P. falciparum* challenge.

### Mosquito behaviour

We used a dual-choice olfactometer^31^ to determine whether changes in body odour throughout *P. falciparum* infection affect mosquito behaviour. Groups of 30 female *An. coluzzii* mosquitoes were given a choice between two traps with either a worn cotton pad or a control cotton pad with ammonia, known to be a mild attractant to *An. coluzzii* ^32^. Mosquito capture rate per worn cotton pad was calculated as a proportion of the total number of mosquitoes caught in both traps (Supplementary Fig. S5).

**Figure 3:**
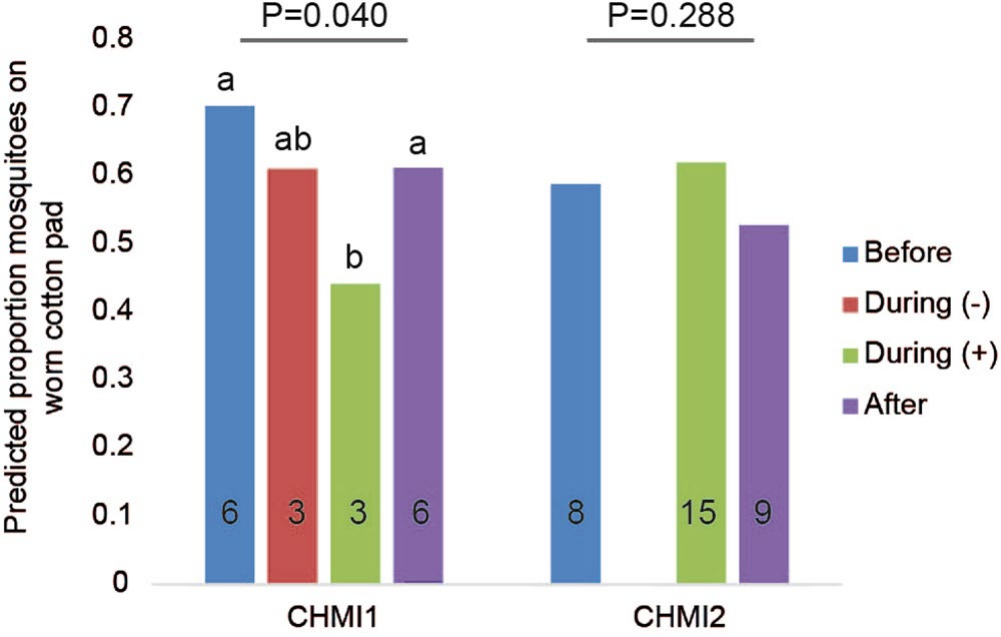
Choice of *Anopheles coluzzii* for cotton pads worn by participants in two Controlled Human Malaria Infection studies. Proportions of mosquitoes trapped in the two traps of the olfactometer are plotted at three sampling time points relative to *P. falciparum* infection: Before, During and After, with parasitological status by qPCR during infection indicated by (−) for qPCR-negatives and (+) for qPCR-positives. Control traps contained a cotton pad with NH_3_. Back-transformed predicted mean proportions of mosquitoes are plotted, from the GLMMs including temporal *P. falciparum* infection as a fixed effect term and participant as a random effect term. Numbers in bars represent the number of cotton pad samples tested in each group (N = 6 individuals in CHMI1, N = 9 individuals in CHMI2). P-values are given for the effect of temporal *P. falciparum* infection within each CHMI study, with significant pairwise differences within CHMI1 indicated by different letters above bars, tested at the logistic scale (P < 0.05). For predicted means (logistic transformation) with standard errors, see Fig. S6, and for pairwise t-probabilities of CHMI1, see Table S3.

In CHIM1, the effect of temporal *P. falciparum* infection was significant (Fig. 3, P = 0.040, GLMM; and see Supplementary Fig. S6 and Table S3). Attractiveness of During (+) samples was significantly lower than that of samples collected before challenge or after antimalarial treatment (P = 0.001 and P = 0.024 respectively, t-probabilities, pairwise comparisons), and During (+) samples were also less attractive than During (−) samples, although this effect was marginally insignificant (P = 0.062). Overall, parasitologically positive samples were significantly less attractive than negative samples (Pos vs. Neg, irrespective of sampling timepoint, P = 0.010, GLMM, Supplementary Fig. S6). Similarly, a reduction in attraction was previously observed in infected mice, which were significantly less attractive than healthy mice 7–8 days after infection^16^. In studies on *Plasmodium*-infected birds, early stage infections did not influence attractiveness of birds to *Culex* mosquitoes^17^, similar to what we found for humans in CHMI2, where relative attractiveness was not affected by temporal parasitological status (Fig. 3, P = 0.28), or parasite presence (Pos vs. Neg, P = 0.18, GLMM, Supplementary Fig. S6).

Contrary to our expectations, both behavioural and chemical data suggest that samples collected after antimalarial treatment were different from samples collected before challenge. Skin odour collected after antimalarial treatment appeared to be less attractive than skin odour collected before malaria challenge, and in CHMI2, the odour profile of After samples separated from odour profiles of the other groups in the CVA (Supplementary Fig. S3), while there was a general trend for greater amounts of volatiles in After compared to Before samples. However, only dodecanal contributed significantly to the difference between After and Before samples (Table 2). In CHMI1, mean amount of RI 1095 was significantly reduced in After samples compared to samples taken at any previous timepoint (Fig. 2a and Table 1). Two participants in CHMI2 that remained parasite-free throughout the study period did not have a different odour profile after antimalarial treatment, supporting our observation that changes in odour profiles after antimalarial treatment result from prior blood stage parasitaemia. *Plasmodium*-induced changes in volatile profile (but not mosquito preference) were also detectable for up to 42 days after challenge in mice, although gametocytes disappeared almost completely after day 18^16^.

Changes in odour profiles varied between the CHMI studies, and were correlated with different effects on attractiveness to *Anopheles* mosquitoes at the early stages of malaria infection. Differences obtained in CHMI1 and CHMI2 may be explained by the number of infectious mosquito bites (five in CHMI1 and one, two or five in CHMI2), immunization and use of chloroquine prophylaxis pre-challenge (respectively in half and all of the participants of CHMI1 and not in CHMI2). Interestingly, in CHMI2, there was evidence that skin odour profiles were differentially influenced by *P. falciparum* strain (NF135.C8 and NF166.C10, Fig. 1c). Individuals in CHMI1 were infected with the more commonly used CHMI strain *P. falciparum* NF54. Peak parasitaemia during the first cycle may be different between these strains, as was shown for NF54 and NF135.C8 in another CHMI study with ten participants^33^. This may have contributed to variation in odour profiles between the two series and would suggest that parasite strains may have distinct and quantitatively different effects on odour profiles and may explain the differences found in mosquito behaviour between the two CHMI studies. Another factor that may have introduced variation not related to *P. falciparum* infection in our study is the inclusion of adult females, as it is known that menstrual cycle can influence odour profile^34^. However, the number of participants in each CHMI, and especially the number of parasitologically positive participants in CHMI1, was too small to disentangle the influence of these factors from the main effect of *P. falciparum* infection.

## Conclusions

In the current study, we observed changes in human skin odour profile during *P. falciparum* infection, on average two days after parasites moved from the liver to the peripheral blood, a phase that is characterized by low densities of asexual parasites^20, 33^ and the absence of gametocytes, as was confirmed. The compounds that were associated with malaria challenged individuals are not known to be produced by *Plasmodium* parasites directly, suggesting that they result from an interaction between the parasite and the host, although the mechanisms leading to changes in odour profile remain unexplained. Increased emission of 2- and 3-methylbutanal and 3-hydroxy-2-butanone that are known to be produced by skin bacteria^29^, may suggest that changes in skin microflora play a role.

With respect to mosquito behaviour, we found reduced attractiveness of parasitologically positive participants in one experiment and no significant effect of *P. falciparum* infection in a second experiment, possibly due to use of different parasite strains. Nevertheless, several identified compounds may be involved in mediating differential attractiveness of *Anopheles* mosquitoes to *P. falciparum* infected humans, including 6-methyl-5-hepten-2-one, 2- and 3-methylbutanal and 3-hydroxy-2-butanone. Such behaviour-modifying compounds can be used in odour-baited mosquito traps that can contribute to vector control^35^.

It is important to follow up the current investigation with studies including later stage infections more likely to harbour gametocytes, in order to understand the impact on transmission and approximate a more natural malaria endemic setting. Our study population consisted of Caucasians naïve to *Plasmodium* infection, who experienced lower densities of asexual parasites and were probably challenged with fewer other infectious organisms than the human population in most malaria endemic areas. Previous findings on Kenyan children and South American adults naturally infected with *P. falciparum* and *P. vivax* respectively, hint at an effect of gametocytaemia on mosquito attraction^11, 12^, but need confirmation in controlled studies with more sensitive diagnostics^36^. This work is currently underway by the authors with Kenyan participants.

## Methods

### Participant population (CHMI)

Participants were recruited from clinical studies at the Radboud University Medical Centre, The Netherlands, during two CHMI trials in 2013 and 2014 [further referred to as CHMI1 and CHMI2, with details of CHMI1 described in ref. 20]. All 17 participants were between 18 and 35 years old (Supplementary methods and Fig. S1 for details).

For both series, odour samples were collected two days prior to *P. falciparum* challenge (further referred to as Before), eight days after challenge (During) and after antimalarial treatment (day 34 post-challenge, After). In CHMI2, additional odour samples were collected six days after *P. falciparum* challenge (i.e. During) from participants that had not yet started antimalarial treatment (Supplementary Fig. S1). Infection was monitored twice daily by thick smear microscopy and by molecular diagnosis [qPCR on 18 S rRNA specific to *P. falciparum* ^37^] from day five post-challenge until antimalarial treatment (Supplementary methods). Additionally, blood samples of CHMI2 participants were assayed by *Pfs25*-qt-NASBA^38^ for the presence of mature gametocytes on the day of antimalarial treatment, on the 3–5 days following treatment, and one week post-treatment.

Ethical approval was obtained from the Dutch Committee on Research Involving Human Subjects (CCMO protocols NL39541.091.12 and NL48704.000.14) and written informed consent was obtained from each participant. Additionally, ethical approval was obtained from the London School of Hygiene and Tropical Medicine (ethics ref: 8510). Sample collection was done in accordance with relevant guidelines and regulations.

### Headspace entrainment and volatile analyses

One foot was placed into a clean bag (Fresh and Eazy oven bags, 45 × 50 cm), which was sealed tightly around the calf using bulldog clips. Charcoal-filtered air was directed into the top of the bag via polytetrafluoroethylene (PTFE) tubing and Swagelok fittings at 1400 ml/min (see supplementary methods for cleaning procedures). The air in the bag was allowed to purge for 30 min via a vacuum pump (700 ml/min), which was connected in a Swagelok fitting at the bottom of the bag without an adsorbent filter. Volatiles were then collected on a 50 mg Tenax filter for 20 min (450 ml/min) and subsequently on a 50 mg Porapak filter for 100 min (650 ml/min). Controls were conducted simultaneously, using the same procedure but with empty bags closed tightly with bulldog clips. Tenax and Porapak filters were immediately placed in clean glass ampoules and sealed under purified nitrogen the following morning (CHMI1), or immediately (CHMI2), before storing at −20 °C until shipping to the London School of Hygiene and Tropical Medicine and Rothamsted Research for chemical analyses.

Porapak filters were eluted with 750 μl re-distilled diethyl ether, and extracts concentrated to 50 μl using a stream of charcoal-filtered nitrogen. Samples were then analysed by gas chromatography (GC, Agilent Technologies, USA; 7890 A for CHMI1 samples, 6890 N and HP6890 for CHMI2 samples; supplementary methods for settings). Traces were integrated (Agilent ChemStation C.01.04) using set integration parameters with an ‘area reject’ equivalent to 0.05 ng, by calibration relative to standard volumes of an alkane series, that was run weekly per GC. Hence, the sensitivity of peak detection was standardized between GC’s. The retention index (RI) and approximate volume of each peak (compound of interest) were calculated relative to the alkanes.

Tenax filters were used specifically to capture and analyse low molecular weight volatiles, as these are masked by the solvent peak used for elution of Porapak samples. Coupled GC-MS was performed on a Micromass Autospec Ultima, magnetic sector mass spectrometer, equipped with a PTV unit (GL Sciences, The Netherlands) and Agilent 6890 N GC (supplementary methods for settings). The software package used was Masslynx V4.1 (Waters, United Kingdom). The Tenax sample chromatograms were manually annotated and peak areas obtained by inspecting peaks between acetone and heptane, respectively eluting from the column between 3 and 11 mins into the program.

Compounds of interest were tentatively identified using coupled GC-MS on five Porapak samples using the same GC-MS procedures, by comparison with MS databases (NIST). Synthetic standards were run for 2-ethylhexanoic acid, 1-dodecene, dodecanal, methyl dodecanoate, 3-hydroxy-2-butanone, 2-methylbutanal and 3-methylbutanal. Elution time and mass spectra of standards were compared to verify identification. No standard of MHO was run because tentative identification based on mass spectra of the peaks of interest was considered conclusive.

### Mosquito behaviour

Following headspace entrainment, two cotton pads (5 cm diameter, 100% cotton, Hema, The Netherlands) were placed on the sole of the foot of each participant^31^, covered with aluminium foil and secured with an island plaster (HEKA®plast border, 10 × 15 cm, Zhejiang Idion Medical Co.Ltd, China; supplementary methods for cleaning procedures).

A dual-port olfactometer, with three identical flight chambers (Tupola, The Netherlands), was used to assess behavioural responses to cotton pads, following methods described previously (e.g.^39^ and with details described in the supplementary methods). Cotton pads from CHMI1 and CHMI2 were tested in separate experiments (Supplementary Fig. S1). The relative attractiveness of samples was obtained by testing a quarter of each worn cotton pad against a quarter of a clean cotton pad with 50 µl of 0.025% ammonia (MERCK, 25% NH_3_, GR for analysis) diluted in water, which is known to be a mild attractant to *An. gambiae s.s*. at low doses^32^. Ammonia was applied approximately 20 min before use in the olfactometer. Cotton pads were placed on stainless steel holders in the centre of the olfactometer trap.

*Anopheles coluzzii* Coetzee & Wilkerson sp. n. (formerly referred to as the M-form of *An. gambiae s.s*. Giles^40^) mosquitoes were used (supplementary methods for rearing procedures). Groups of 30 female mosquitoes (5–8 days old) were released at the downwind end of each flight chamber, and allowed to fly for 15 min. The number of mosquitoes that had entered the traps was then counted. The relative attractiveness of each cotton pad was established by repeating the trials six times with 30 mosquitoes on different experimental days (supplementary methods for randomization).

### Statistical analyses

Samples were categorized by sampling timepoint and parasitological status. Samples collected before challenge (Before) or after antimalarial treatment (After) were all qPCR-negative. Samples taken during infection were grouped into During (+) when they had at least one positive qPCR-result on the day of headspace entrainment for chemical analyses, and/or on the following morning for behavioural analyses because cotton pads were worn overnight. The During (−) group contains samples that were qPCR-negative during infection (Supplementary Fig. S1).

The total amount of each compound produced during the 100 min entrainment on Porapak was compared between samples. Canonical variate analysis (CVA) was used to allow comparisons of multiple compounds across different groupings. Porapak samples from CHMI1 and CHMI2 were analysed separately using the four categories described above (Before, During (−), During (+), After) as groupings in addition to empty bag and ether solvent controls. In an additional analysis, to maximize sample size in CVA, samples were grouped by parasitological status only (positive, negative, empty bag control and ether control). For each CVA, the magnitude of the loadings on each compound within canonical variate (CV) 1 and CV2 was inspected to determine which compounds contributed most to observed differences between group means. Within a CV, compounds with corresponding loadings greater than half the maximum observed loading were selected. The mean volumes of these compounds were compared per group, paying consideration to the volume produced per individual sample. For Tenax samples, the mean quantity of each compound was examined per group in a similar way. This resulted in a list of compounds of interest (COI) per series, for further statistical examination.

COI were analysed individually using linear mixed models, fitted using the method of residual maximum likelihood (REML). Samples of two participants in CHMI2 were excluded from this analysis because they did not become positive for *P. falciparum* infection at any timepoint and differences in baseline levels of volatile emission could have affected our conclusions. This model tested (F-tests) for the overall effect of status [Before, During (−), During (+), After], whilst accounting for the repeated observations on participants and unequal numbers of observations per participant in CHMI2. Pairwise comparisons between predicted means from the model were done using least significant differences at the 5% level of significance. Total volume (ng) of all compounds detected on Porapak samples was also compared between parasitologically positive and negative samples per CHMI using REML.

We also tested the effect of temporal parasitological status [categories Before, During (−), During (+) and After] on attractiveness of worn cotton pads relative to NH_3_ controls, for each CHMI separately. Logistic linear regression models were used with a Binomial distribution and logit link function. In the generalized linear mixed models (GLMMs), the participants were taken into account as a random effect term, and the effect of *P. falciparum* infection was tested as a fixed effect term. Mosquito numbers trapped on each odour sample (worn cotton pad) were summed over six replicates, and used as the response variable, while the total number of mosquitoes in both traps summed over six replicates per sample was used as the Binomial total, thus taking into account that estimates based on larger samples are more precise. Dispersion was estimated to account for overdispersion. Pairwise t-probabilities were obtained to test for differences between the four categories of *P. falciparum* infection states on the logistic scale.

We also used a GLMM to test the effect of parasitological status (positive or negative by qPCR) on attractiveness of worn cotton pads, irrespective of sampling timepoint, to maximize sample size.

Genstat^®^ (2015, 17th edition, ©VSN International, Hemel Hempstead, UK) was used for the analyses. All data analysed during this study are included in the Supplementary Information files of this published article.

## Electronic supplementary material

Supplementary information

Dataset 1A

Dataset 1B

Dataset 2A

Dataset 2B

Dataset 2C

Dataset 2D

Dataset 3
