# Supplementary material for: Odours of Plasmodium falciparum-infected participants influence mosquito-host interactions
Source: Sci Rep. 2017 Aug 24;7:9283. doi: 10.1038/s41598-017-08978-9 (PMC5570919; doi:10.1038/s41598-017-08978-9)
Supplement: Supplementary file 1 — Supplementary information [file 41598_2017_8978_MOESM1_ESM.pdf]

Supplementary material

Odours of *Plasmodium falciparum*-infected participants influence mosquito-host interactions

Jetske G. de Boer[a], Ailie Robinson[b], Stephen J. Powers[c], Saskia L.G.E. Burgers[d], John C. Caulfield[e], Michael A. Birkett[e], Renate C. Smallegange[a,b], Perry J.J. van Genderen[f], Teun Bousema[g], Robert W. Sauerwein[g], John A. Pickett[e], Willem Takken[a], James G. Logan[b]

Supplementary methods

Participant population

Participants in the clinical studies were given the choice of whether or not to take part in the 'odour profile' sub-study if they met the additional inclusion criteria, i.e. not being a regular smoker and no regular use of medication. For 24 h. prior to odour collection, and 12 h. after commencement of measurements, participants were asked to abstain from the consumption of alcohol, garlic, onion, potent herbs and spices, not to shower and to abstain from the use of perfumed cosmetics. They were also provided with non-perfumed soap (Neutral® shower gel and shampoo, Unilever, The Netherlands), for use during the last shower before this period.

In CHMI1, half of the study population were inoculated with four doses of a test sporozoite vaccine and half with saline solution. All participants received chloroquine chemoprophylaxis simultaneously for 14 weeks (starting with a loading dose of 300 mg on each of the first two days, followed by a weekly dose of 300 mg chloroquine), and for an additional period of six weeks after approximately five months. Challenge with *P. falciparum* sporozoites of the Pf NF54 strain by the bites of five infectious mosquitoes (*An. stephensi*) took place 33 days after the last dose of chloroquine. In CHMI2, participants were exposed to the bites of one, two or five mosquitoes infected with *P. falciparum* sporozoites of either the Pf NF135.C8 or Pf NF166.C10 strain. No individuals were immunized or treated with antimalarials prior to challenge. Subjects were treated via a curative regimen of Malarone® (each tablet containing 250 mg atovaquone and 100 mg proguanil) following two consecutive positive qPCR results, or at day 21 (CHMI1) or day 13 (CHMI2) post-challenge.

Cleaning procedures for headspace entrainment and cotton pads

Prior to use, all PTFE tubing was cleaned by running 70 % ethanol through and baking in an oven at 150 °C for two h. Bags were cleaned by baking in the oven at the same temperature and duration, and all Swagelok fittings were sprayed with ethanol and baked prior to use. Charcoal filters were cleaned at least once a week by emptying the charcoal onto an aluminium tray and baking at 150 °C for two h. Glassware was cleaned with 70 % ethanol and acetone, and then baked. Clean cotton gloves were worn by the investigators throughout the entrainment process.

Prior to use, the cotton pads were cleaned in a clean glass Petri dish with hexane (MERCK, >96%) for one min, after which excess liquid was squeezed out and they were placed in another Petri dish with methanol (MERCK, >99.5%) for one min. Finally they were placed in clean glass vials and baked at 100°C for 2 h.

Mosquito colony maintenance

The colony of *Anopheles coluzzii* Coetzee & Wilkerson sp. n. (formerly referred to as the M-form of *An. gambiae s.s.* Giles, [1]) used originated from Suakoko, Liberia, and has been kept at the laboratory of Entomology in Wageningen since 1987. Mosquitoes were fed on human blood (Sanquin Blood Supply Foundation, Nijmegen, The Netherlands) daily using a membrane feeding system (Hemotec® PS5, Discovery Workshops, UK) covered with Parafilm® and set at 38°C. A sock releasing human odour and 5% CO2 were offered during blood-feeding to expose mosquitoes to natural host-derived cues. Adult mosquitoes were kept in 30-cm cubic gauze-covered cages (Bugdorm®, Megaview, Taiwan) in a climate-controlled room (27±1°C, 80±5% RH, LD 12:12) and had access to 6% (w/v) glucose solution offered on filter paper. Larvae were reared on Liquifry No. 1 (Interpet, Dorking, U.K.) and Tetramin® baby fish food (Tetra GmbH, Melle, Germany) and pupae were collected daily. Groups of 30 females (5-8 days old and

presumably mated but not blood-fed) were collected approximately 16-22 h. before experiments and provided with tap water on damp cotton wool.

Dual-port olfactometer

In the dual-port olfactometer, charcoal-filtered, moisturized air was blown into poly-methyl-methyl-acrylate traps, which held the odour samples (cotton pads, see below). Data loggers (MSR145S, MSR Electronics, GmBhm, Switzerland) were used to monitor temperature, humidity and air pressure in the room, and in each of the three flight chambers. Air entered the traps at a temperature of 29±0.5°C, and humidity of more than 80%, and was released into the flight chambers at a speed of approximately 0.15-0.25 m/s. CO_2 (5%) was released below the entrance to each trap at a rate of 175 ml/min in all trials. Temperature inside the flight chambers was 26.5±0.5°C, and humidity inside the flight chambers was 73±5°C in series 1, and 66±5°C in series 2. A dim light (<1 lux) was on during trials but the room was otherwise kept dark. Experiments were performed during the last four h. of the scotophase, when *An. gambiae s.l.* is known to be highly responsive to host odour (e.g. [2]).

Mosquitoes that remained in the flight chamber after 15 min were removed with a vacuum cleaner before starting the next trial. Mosquitoes that remained in the release cages were counted and removed. Each trial started with new groups of mosquitoes, clean traps and odour samples with the exception of worn cotton pads that were re-used a maximum of three times on different days and frozen at -20°C between experiments. This level of replication does not lead to depletion of skin odour present on the cotton pads [3]. Cotton pads with ammonia were only used once.

Over the six replicates, the sequence of worn cotton pads was randomized in such a way that each sample was tested once in each position of the olfactometer, i.e. on the left and right side of each of the three flight chambers. The total number of samples in CHMI2 was too high to test them all on the same day, so we divided participants into two groups. Groups of participants were randomized between experimental replicates so that a particular participant was tested on the same day as each of the other participants at least once. Cotton pads worn by the same participant at different time points (i.e. Before, During and After) were always tested on the same experimental day.

Settings for GC and GC-MS

High resolution GC analysis was carried out using Agilent GC instruments that were fitted with a cool-on-column injector, flame ionization detector, non-polar HP1 column (50 m x 0.32 mm with film thickness of 0.52 µm) and used hydrogen as carrier gas. The oven temperature was maintained at 40 °C for 0.5 min and then programmed to increase by 5 °C per min to 150 °C, held for 0.1 minute and then raised by 10 °C per minute to 230 °C, where it was held for 40 min.

For coupled GC-MS analysis, a Waters Autospec Ultima instrument (Manchester, UK) was fitted with a non-polar HP1 column (50m length x 0.32mm and 0.52µm film thickness, J & W Scientific). Ionization was by electron impact (70 eV, 220°C). The GC oven temperature was maintained at 30°C for 5 min and then programmed to rise at 5°C/min to 250°C. Samples were analysed by thermal desorption (PTV unit programmed to start at 30°C then to rise to 250°C at 16°C/sec). The carrier gas was helium.

Supplementary figures

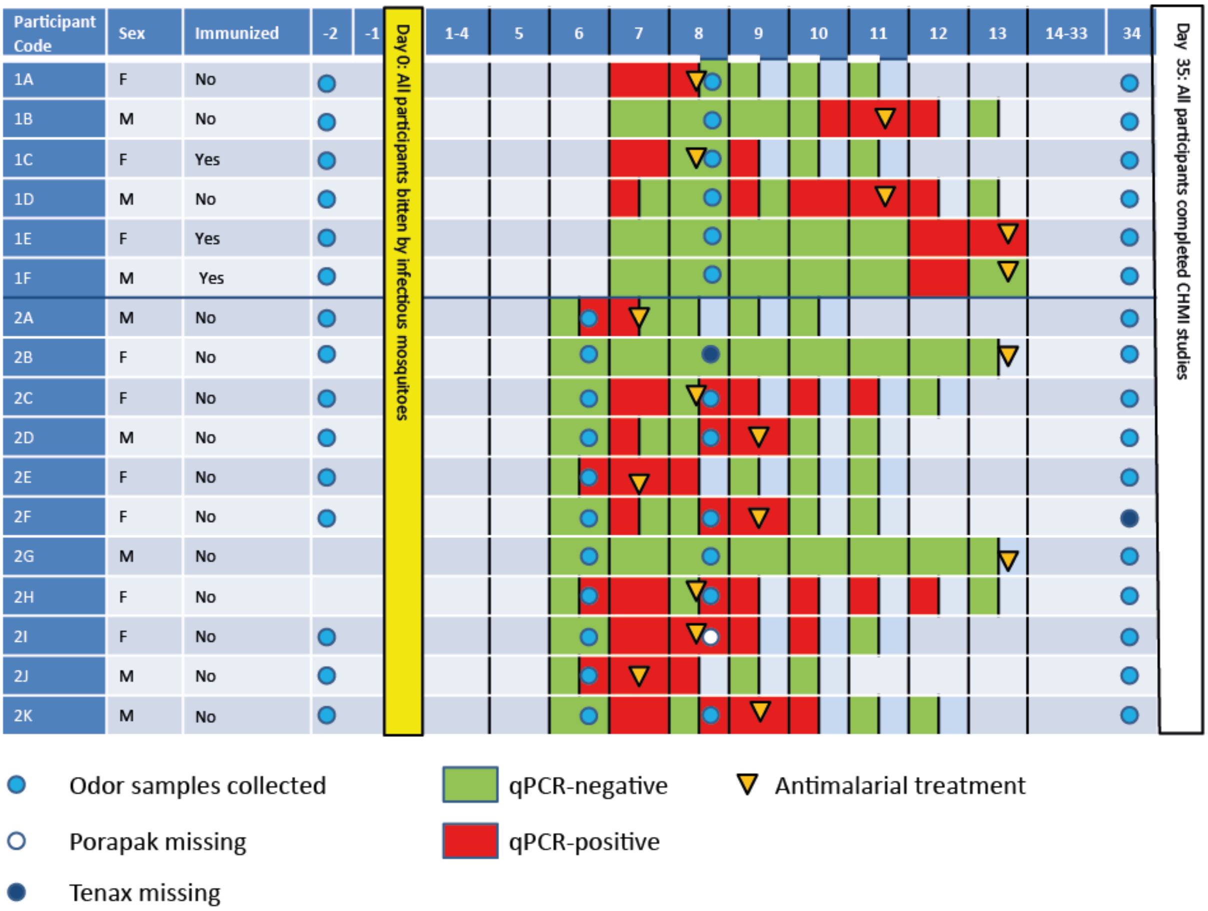

Figure S1. Overview of participants in the clinical malaria studies. CHMI1 had six participants (1A-1F), and CHMI2 had 11 participants (2A-2K). On day 0, participants were challenged with *P. falciparum* strain NF54 (CHMI1), NF135.C8 or NF166.C10 (CHMI2) by bites of infectious *An. stephensi* mosquitoes (5 mosquitoes per participant in CHMI1, and 1, 2 or 5 mosquitoes in CHMI2). For each participant, sex and immunization status are indicated. Parasitological status was monitored twice per day by qPCR between days 7 to 13 post challenge for CHMI1, and between days 6 and 13 for CHMI2; red squares refer to qPCR-positives and green squares to qPCR-negatives. Antimalarial treatment was started following two subsequent positive qPCRs, and is indicated with orange triangles. Sampling time points for air entrainment of foot headspace are indicated by circles for each participant; cotton pads were collected at the same time points overnight. In CHMI1, two participants received treatment on the afternoon just prior to headspace entrainment (1A and 1C). They were considered positive in all analyses because they had two consecutive positive qPCR-results prior to headspace entrainment and cotton pad collection. The third participant in this group that was considered positive (1D) had a positive qPCR in the mornings of the days before and after headspace entrainment. It should be noted that these three participants were qPCR-negative on the evening of headspace entrainment. In CHMI2, two participants received treatment during headspace entrainment and one just after headspace entrainment (while wearing the cotton pad) (2C, 2H and 2I). Of these, participant 2I had a fever at this time point. All three had positive qPCR-results on the evening of headspace entrainment and on the following morning and were thus considered positive in all analyses. Five participants became positive for the first time in the

morning of day 7 post challenge, and were thus considered positive for behavioural analyses but negative for analyses of the odour profile.

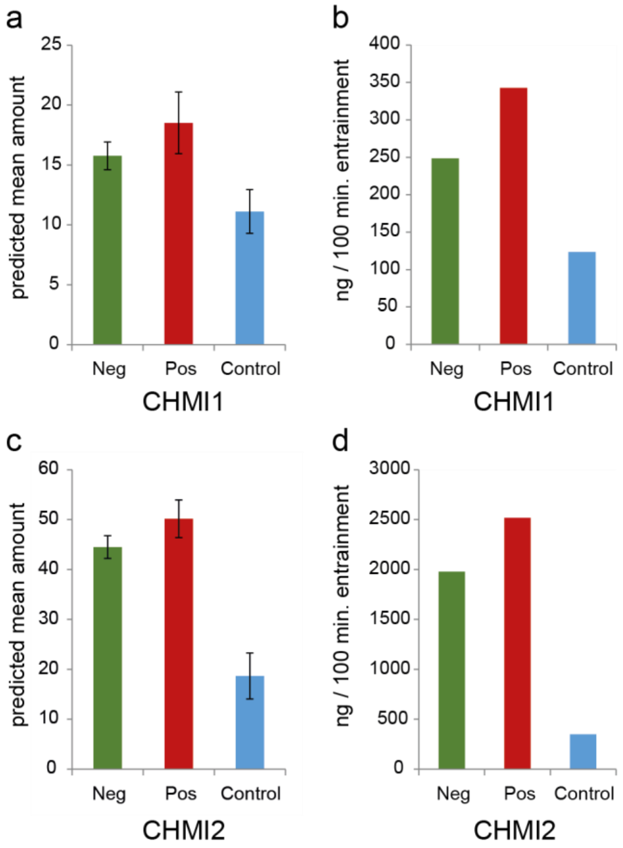

Figure S2. Total volume of compounds produced in samples of parasitologically positive and negative individuals and (empty bag) controls. All compounds examined in the analyses of Porapak samples collected over the duration of 100 min. entrainment were compared in CHMI1 (panels A and B) and CHMI2 (panels C and D). Panels A and C show predicted mean amounts (logistic transformation) and standard errors for sum of all compounds, derived from linear mixed models, which were fitted using the method of residual maximum likelihood (P=0.052 for CHMI1, pos vs. neg not significant, LSD=5.87, actual difference=2.75; P<0.001 for CHMI2, pos vs. neg not significant, LSD=8, actual difference=5). Back transformed amounts (ng) for sum of all compounds are shown in panels B and D. Number of samples in CHMI1 (with the number of individuals in brackets): Pos n=3 (3), Neg n=15 (6), Control n=6; in CHMI2: Pos n=9 (8), Neg n=29 (11), Control n=6.

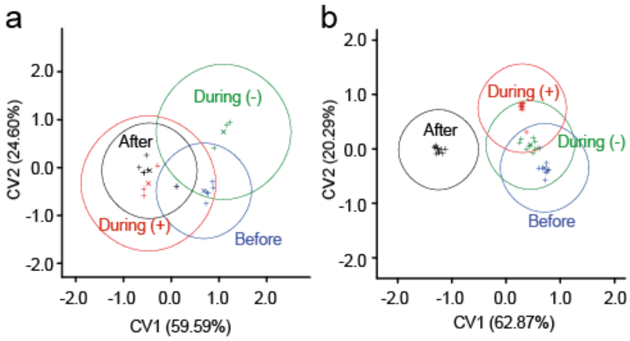

Figure S3. Canonical variates plots of chemistry data (Porapak samples). Four groups were used to test for the combined effect of sampling time point and parasitological status. Samples are positioned by scores (indicated by + symbols) on two canonical variates axes relative to the presence and quantity of compounds contributing to these axes. The percentage of possible discrimination accounted for is included in the axes labels. Circles represent 95 % confidence intervals around the means (× symbols) of scores. A, Number of samples for CHMI1: Before n=6, During (-) n=3, During (+) n=3, After n= 6. B, CHMI2 (number of individuals in brackets): Before n=9 (9), During (-) n=9 (7), During (+) n=9 (8), After n=11 (11). Controls of empty bag sampling and the di-ethyl ether used during sample elution were analysed and included as control groups but excluded from figures to maintain clarity. Parasitological status was defined by qPCR-testing on the same day.

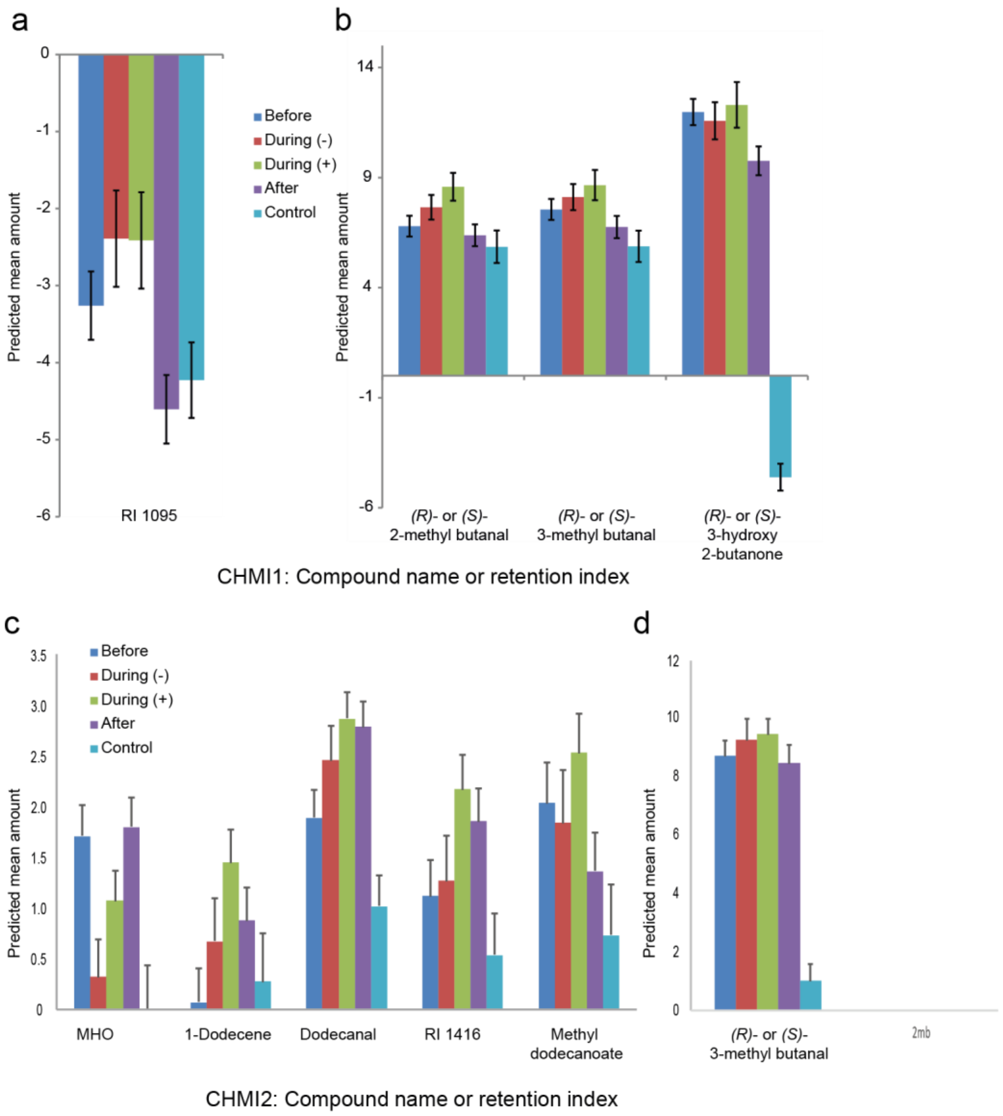

Figure S4. Levels of compounds of interest identified in samples of CHMI1 (A and B) and CHMI2 (C and D). Levels of compounds were analysed by temporal parasitological status (Before: sampling time point before malaria challenge; During: 6 and/or 8 days post challenge, with (–) and (+) referring to parasitological status, measured by qPCR; After: after antimalarial treatment; Control: headspace collected from empty control bags). Predicted means (log transformation) with standard errors are shown derived from the REML with compounds collected on Porapak adsorbent in panels A and C, and compounds collected on Tenax adsorbent in panels B and D. Number of Porapak and Tenax samples in CHMI1: Before n=6, During (-) n=3, During (+) n=3, After n=6, Control n=6. See Supplementary Table S1 for pairwise SEDs. Number of samples in CHMI2 (number of individuals in brackets): Porapak – Before n=8 (8), During (-) n=5 (5), During (+) n=9 (8), After n=9 (9), Control n=6; Tenax – Before n=8 (8), During (-) n=4 (4), During (+) n=10 (9), After n=9 (9), Control n=6. RI 1095 (CHMI1) and 1-Dodecene, dodecanal, methyl dodecanoate and RI 1416 (CHMI2) each co-eluted with another unidentified compound and hence their quantities are difficult to obtain accurately. Differences in amounts observed in temporal *P. falciparum* infection categories can therefore not be attributed with absolute certainty to these compounds. RI 1095 contained *(R)*- or *(S)*-2-ethylhexanoic acid and an unidentified compound, and RI 1416 contained an unidentified sesquiterpene and a phthalate. See Supplementary Table S2 for pairwise SEDs.

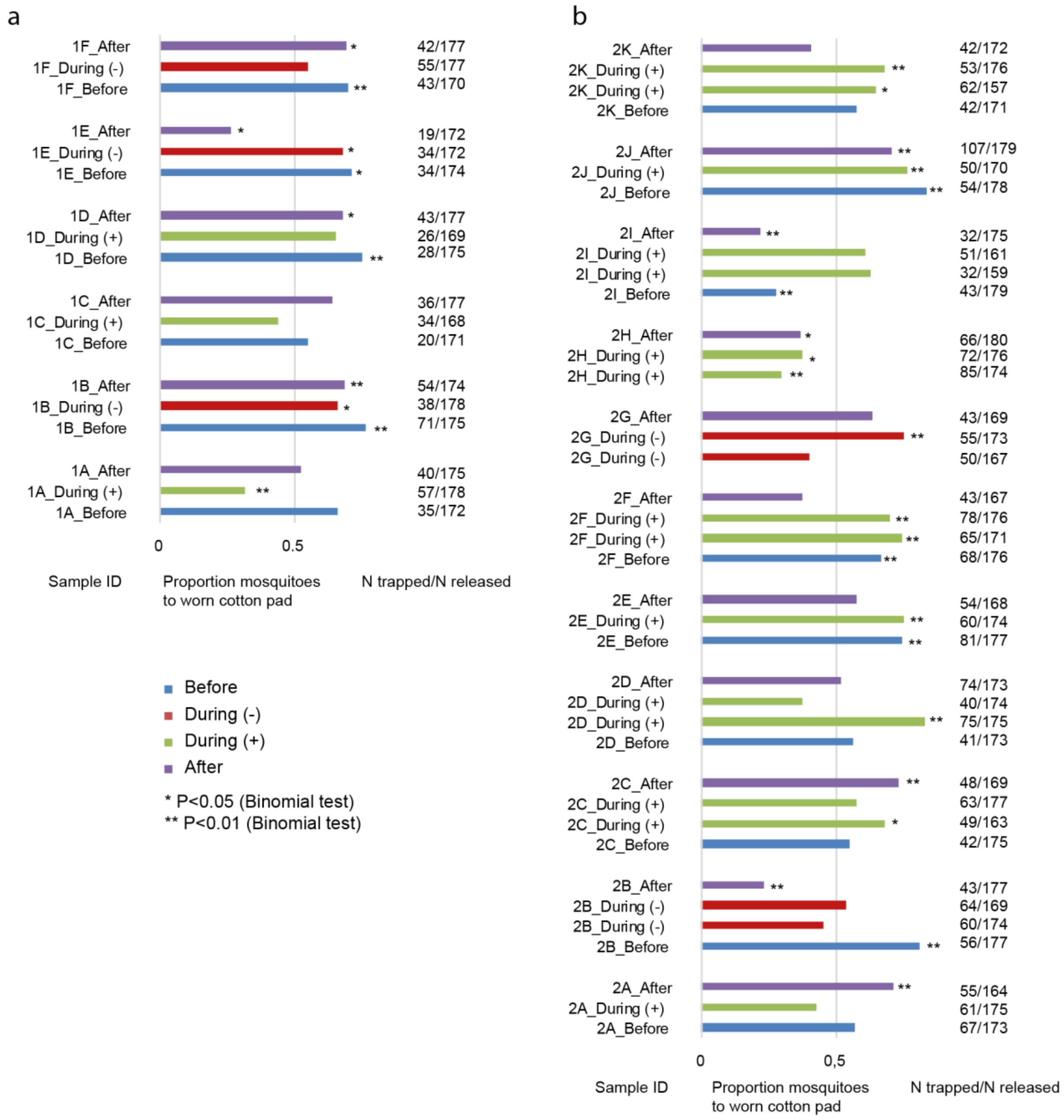

Figure S5. *Anopheles coluzzii* responses to individual cotton pads worn by participants, summed over 6 replicates in the olfactometer. Attractiveness of worn cotton pads is reported as the proportion of mosquitoes that chose the worn cotton pad relative to the total number of mosquitoes trapped (panel A, CHMI1; panel B, CHMI2), i.e. 0.5 indicates that an equal number of mosquitoes was trapped on the worn cotton pad as on the control. Participant code, sampling time point and infection status indicated on the left side as the sample ID. The total number of mosquitoes trapped (N trapped), and the number of mosquitoes released is shown on the right side. Significant preference for the worn cotton pad or NH_3 is indicated for each bar (two-sided Binomial tests: * $P<0.05$; ** $P<0.01$). Binomial tests were done using the total number of mosquitoes trapped on the worn and control cotton pads over six replicates, with the null hypothesis that the proportion trapped on the worn cotton pad was 0.5.

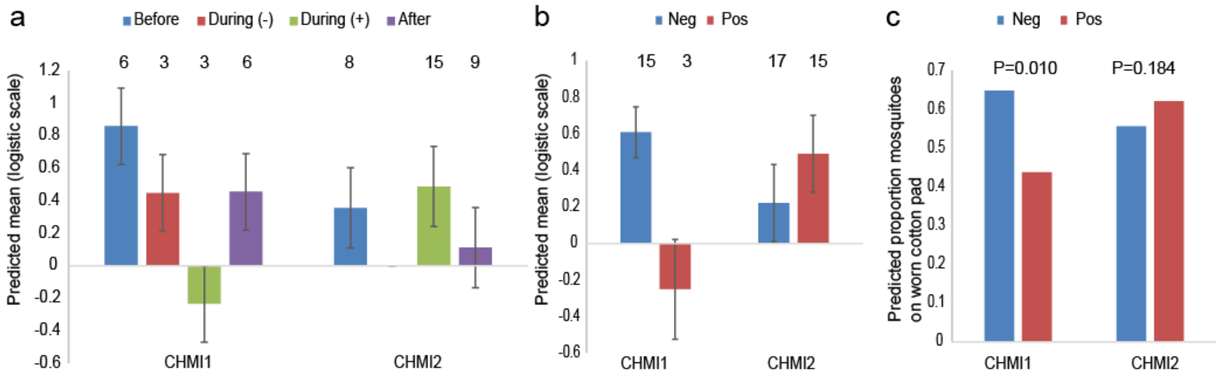

Figure S6. Relative choice of *Anopheles coluzzii* for worn cotton pads in the olfactometer. Panel A shows analysis by temporal parasitological status [Before, During (-), During (+) and After], with predicted mean proportions of mosquitoes trapped on the worn cotton pads over six replicates plotted on the logistic scale with average standard errors derived from the generalized linear mixed models including parasitological status as a fixed effect term (P=0.030 in CHMI1, P=0.031 in CHMI2) and participant as a random effect term. For matrices of pairwise SEDs, see Supplementary Table S3. Panels B and C show analyses by parasitological status irrespective of sampling time point in both CHMI studies. Predicted mean proportions with respective standard errors are plotted on the logistic scale in panel B, and back-transformed predicted mean proportions of mosquitoes are plotted in panel C, from the GLMMs including parasitological status as a fixed effect term and participant as a random effect term. Numbers above columns in panels A and B represent the number of cotton pad samples tested in each group.

Supplementary tables

Table S1. Matrices of pairwise comparisons between temporal infection groups [Before, During (-), During (+), After, and Control, i.e. headspace from empty bag] for compounds of interest identified in CHMI1. Standard errors of differences between groups are shown, as derived from the linear mixed models (REML), and compound names or retention indices provided. Significance (P< 0.05) for comparisons made using the least significant difference (LSD, 5 %) values (= SED multiplied by the 2.5 % t-value) is indicated by a + symbol.

Unidentified (RI 1095, includes *(R)*- or *(S)*-2-ethylhexanoic acid)

**Category (parasitology)**	**Standard error of differences**				
Before	*				
During (-)	0.7390	*			
During (+)	0.7390	0.8797	*		
After	0.5938 +	0.7390 +	0.7390 +	*	
Control	0.6617	0.7946 +	0.7946 +	0.6617	*
df = 30	Before	During (-)	During (+)	After	Control

(R)- or *(S)*-2-methylbutanal

**Category (parasitology)**	**Standard error of differences**				
Before	*				
During (-)	0.46	*			
During (+)	0.5459 +	0.6571	*		
After	0.3775	0.4636 +	0.5489 +	*	
Control	0.8773	0.9273	0.9727 +	0.8892	*
df = 11	Before	During (-)	During (+)	After	Control

(R)- or *(S)*-3-methylbutanal

**Category (parasitology)**	**Standard error of differences**				
Before	*				
During (-)	0.5477	*			
During (+)	0.6487	0.7773	*		
After	0.4509	0.5538 +	0.6539 +	*	
Control	0.8569	0.9269 +	0.9899 +	0.8732	*
df = 11	Before	During (-)	During (+)	After	Control

(R)- or *(S)*-3-hydroxy-2-butanone

**Category (parasitology)**	**Standard error of differences**				
Before	*				
During (-)	1.03	*			
During (+)	1.191	1.337	*		
After	0.88 +	1.063	1.219	*	
Control	0.853 +	1.041 +	1.201 +	0.894 +	*
df = 12	Before	During (-)	During (+)	After	Control

Table S2. Matrices of pairwise comparisons between temporal infection groups (Before, During (-), During (+), After, and Control) for the compounds of interest identified in CHMI2. Standard errors of differences between groups are shown, as derived from linear mixed models (REML), and compound names or retention indices given. Significance (P< 0.05) for comparisons made using the least significant difference (LSD, 5 %) values (= SED multiplied by the 2.5 % t-value) is indicated by a + symbol.

6-methyl-5-hepten-2-one (RI 972)

**Category (parasitology)**	**Standard error of differences**				
Before	*				
During (-)	0.434 +	*			
During (+)	0.372	0.435	*		
After	0.365	0.427 +	0.356	*	
Control	0.525 +	0.569	0.518 +	0.516 +	*
df = 24	Before	During (-)	During (+)	After	Control

1-Dodecene (RI 1193)

**Category (parasitology)**	**Standard error of differences**				
Before	*				
During (-)	0.500	*			
During (+)	0.428 +	0.499	*		
After	0.421	0.491	0.410	*	
Control	0.584	0.636	0.576	0.574	*
df = 24	Before	During (-)	During (+)	After	Control

Dodecanal (RI 1392)

**Category (parasitology)**	**Standard error of differences**				
Before	*				
During (-)	0.433	*			
During (+)	0.369 +	0.423	*		
After	0.369 +	0.423	0.358	*	
Control	0.410 +	0.459 +	0.400 +	0.400 +	*
df = 32	Before	During (-)	During (+)	After	Control

Unidentified sesquiterpene (RI 1416)

**Category (parasitology)**	**Standard error of differences**				
Before	*				
During (-)	0.570	*			
During (+)	0.486 +	0.557	*		
After	0.486	0.557	0.471	*	
Control	0.540	0.605	0.527 +	0.527 +	*
df = 32	Before	During (-)	During (+)	After	Control

Methyl dodecanoate (RI 1509)

**Category (parasitology)**	**Standard error of differences**				
Before	*				
During (-)	0.655	*			
During (+)	0.559	0.644	*		
After	0.557	0.642	0.541 +	*	
Control	0.641	0.716	0.627 +	0.626	*
df = 24	Before	During (-)	During (+)	After	Control

(R)- or *(S)*-3-methylbutanal

**Category (parasitology)**	**Standard error of differences**				
Before	*				
During (-)	0.895	*			
During (+)	0.739	0.874	*		
After	0.836	0.958	0.814 +	*	
Control	0.794 +	0.921 +	0.771 +	0.864 +	*
df = 24	Before	During (-)	During (+)	After	Control

Table S3. Matrix of pairwise comparisons between temporal infection status groups [Before, During (-), During (+), After] for the proportions of mosquitoes attracted to worn cotton pads in the olfactometer for CHMI1. T-probabilities for pairwise comparisons between groups are shown, following the generalized linear mixed model (GLMM). Significant pairwise differences (P<0.05) are indicated with +.

CHMI1, P=0.040 (GLMM)

**Category (parasitology)**	**Standard error of differences**			
Before	*			
During (-)	0.180	*		
During (+)	0.001 +	0.062	*	
After	0.114	0.981	0.024 +	*
	Before	During (-)	During (+)	After

References

1 Coetzee, M. *et al.* *Anopheles coluzzii* and *Anopheles amharicus*, new members of the *Anopheles gambiae* complex. *Zootaxa* **3619**, 246-274, doi:10.11646/zootaxa.3619.3.2 (2013).

2 Killeen, G. F. *et al.* Quantifying behavioural interactions between humans and mosquitoes: Evaluating the protective efficacy of insecticidal nets against malaria transmission in rural Tanzania. *BMC Infect. Dis.* **6**, doi:10.1186/1471-2334-6-161 (2006).

3 Verhulst, N. O., Weldegergis, B. T., Menger, D. & Takken, W. Attractiveness of volatiles from different body parts to the malaria mosquito *Anopheles coluzzii* is affected by deodorant compounds. *Sci. Rep.* **6**, doi:10.1038/srep27141 (2016).
